# 1-Carboxy­methyl-2-ethyl-4-methyl-1*H*-imidazol-3-ium chloride monohydrate

**DOI:** 10.1107/S1600536809013403

**Published:** 2009-04-22

**Authors:** Chuan-Qing Chen, Shi-Neng Luo, Jian-Guo Lin, Ling Qiu, Yong-Mei Xia

**Affiliations:** a School of Chemical and Materials Engineering, Jiangnan University, Wuxi 214122, People’s Republic of China; b The Key Laboratory of Nuclear Medicine, Ministry of Health, Jiangsu Institute of Nuclear Medicine, Wuxi, 214063, People’s Republic of China

## Abstract

In the title compound, C_8_H_13_N_2_O_2_^+^·Cl^−^·H_2_O, the methyl C atom of the ethyl group is slightly out of the imidazole plane, with an N—C(ring)—C—C torsion angle of −15.1 (2)°. In the crystal structure, there are strong inter­molecular hydrogen-bonding inter­actions between the solvent water mol­ecule, the free chloride anion and the organic cation, resulting in a two-dimensional supra­molecular network in the *ab* plane.

## Related literature

The title compound is a vital intermediate in the synthesis of bisphosphonic acid, *i.e.* 2-(2-ethyl-4-methyl-1*H*-imidazol-1-yl)-1-hydroxyethane-1,1-diyldiphosphonic acid; for a general background on bis­phospho­nates, see: Dawson (2003 ▶); Vasireddy *et al.* (2003 ▶). For related structures, see: Gao *et al.* (2004 ▶); Barczynski *et al.* (2008 ▶). For the synthesis, see: Zederenko *et al.* (1994 ▶).
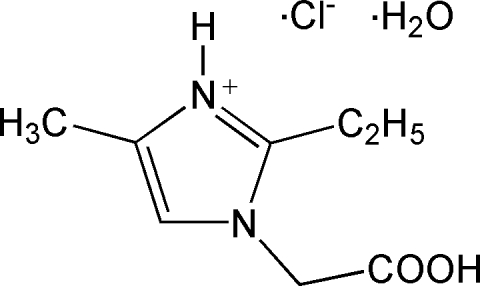


## Experimental

### 

#### Crystal data

C_8_H_13_N_2_O_2_^+^·Cl^−^·H_2_O
*M* *_r_* = 222.67
Monoclinic, 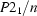

*a* = 11.077 (2) Å
*b* = 8.4542 (18) Å
*c* = 11.938 (3) Å
β = 90.265 (3)°
*V* = 1117.9 (4) Å^3^
*Z* = 4
Mo *K*α radiation
μ = 0.33 mm^−1^
*T* = 93 K
0.40 × 0.40 × 0.35 mm

#### Data collection

Rigaku SPIDER diffractometer
Absorption correction: multi-scan (*RAPID-AUTO*; Rigaku, 2004 ▶) *T*_min_ = 0.880, *T*_max_ = 0.894
8869 measured reflections
2532 independent reflections
2203 reflections with *I* > 2σ(*I*)
*R*_int_ = 0.031

#### Refinement

*R*[*F* ^2^ > 2σ(*F* ^2^)] = 0.036
*wR*(*F* ^2^) = 0.099
*S* = 1.00
2532 reflections
145 parameters
1 restraint
H atoms treated by a mixture of independent and constrained refinement
Δρ_max_ = 0.29 e Å^−3^
Δρ_min_ = −0.20 e Å^−3^

### 

Data collection: *RAPID-AUTO* (Rigaku, 2004 ▶); cell refinement: *RAPID-AUTO*; data reduction: *RAPID-AUTO*; program(s) used to solve structure: *SHELXS97* (Sheldrick, 2008 ▶); program(s) used to refine structure: *SHELXL97* (Sheldrick, 2008 ▶); molecular graphics: *SHELXTL* (Sheldrick, 2008 ▶); software used to prepare material for publication: *SHELXTL*.

## Supplementary Material

Crystal structure: contains datablocks global, I. DOI: 10.1107/S1600536809013403/fj2205sup1.cif

Structure factors: contains datablocks I. DOI: 10.1107/S1600536809013403/fj2205Isup2.hkl

Additional supplementary materials:  crystallographic information; 3D view; checkCIF report

## Figures and Tables

**Table 1:** Hydrogen-bond geometry (Å, °)

*D*—H⋯*A*	*D*—H	H⋯*A*	*D*⋯*A*	*D*—H⋯*A*
N1—H1*N*⋯Cl1	0.881 (17)	2.300 (18)	3.1635 (14)	166.6 (15)
O3—H3*A*⋯Cl1	0.91 (2)	2.20 (2)	3.1062 (14)	177 (2)
O1—H1*O*⋯O3^i^	0.96 (2)	1.60 (2)	2.5557 (16)	170 (2)
O3—H3*B*⋯Cl1^ii^	0.96 (2)	2.14 (2)	3.0860 (14)	168.2 (19)

Symmetry codes: (i) 
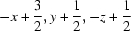
; (ii) 
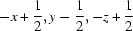
.

**Fig. 1.**
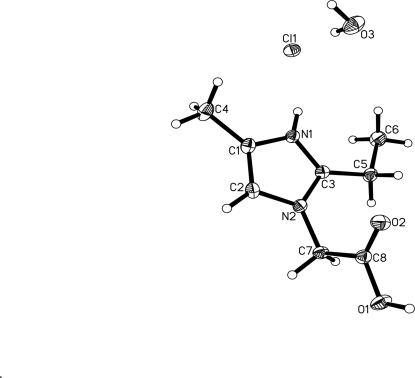
The molecular structure of (I), with atom labels and 30% probability displacement ellipsoids for non-H atoms.

**Fig. 2.**
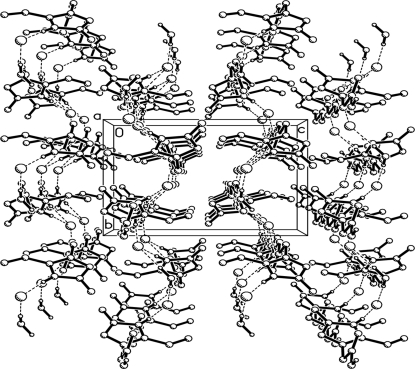
Perspective view of the supramolecular network built from strong intermolecular hydrogen bonds (dashed lines). H atoms not involved in hydrogen bonds have been omitted.

## supplementary crystallographic information

### Comment

Bisphosphonates with an imidazole ring, namely zoledronate, are effective bone-specific palliative treatments that reduce tumor-induced skeletal complications. With this idea in mind, we intend to synthesis one of the third-generation bisphosphonate compound, 2-(2-ethyl-4-methyl-1*H*-imidazol-1-yl)-1–1-hydroxyethane-1,1- bisphosphonic acid, which is potentially used for treatment of patients. As a vital intermediate compound for the stepwise reactions of the bisphosphonic acid, the synthesis and crystal structure of the title compound has been reported herein.

In the title compound (I) (Fig. 1), C_8_H_13_N_2_O_2_^+^.Cl^-^.H_2_O, all the carbon atoms (C4, C5 and C7) linked to the imidazole ring are almost coplanar with the imidazole ring. The ethyl is slightly out of the imidazole plane with an N1—C3(ring)-C5—C6 torsion angle of -15.116 (211)°. While the 1-substituted acetic acid group is approximately perpendicular to the imidazole ring [dihedral angle = 77.438 (111)°]. There are strong intermolecular hydrogen interactions between the free water molecule (O3), the free chloride anion (Cl1), and the O1 and N1 from the organic cation (Table 1). And the crystal structure is stabilized by these strong hydrogen bond interactions to form two-dimensional supramolecular network along *ab* plane (Table 1 and Fig. 2).

### Experimental

The title compound (I) was synthesized according to previous literature (Zederenko *et al.*, 1994). After reaction, a white powder was obtained (yield 65%). Mp 170–171 °C. Then, compound (I) was recrystallized from acetone solvent; colourless block-shaped crystals were formed after several days (yield 61%). Analysis calculated for C_8_H_15_ClN_2_O_3_: 43.15, H 6.79, N 12.58%; found: C 43.01, H 6.96, N 12.45%.

### Crystal data

**Table:**

C_8_H_13_N_2_O_2_^+^·Cl^−^·H_2_O	*F*(000) = 472
*M**_r_* = 222.67	*D*_x_ = 1.323 Mg m^−^^3^
Monoclinic, *P*2_1_/*n*	Mo *K*α radiation, λ = 0.71073 Å
Hall symbol: -P 2yn	Cell parameters from 3544 reflections
*a* = 11.077 (2) Å	θ = 3.0–27.5°
*b* = 8.4542 (18) Å	µ = 0.33 mm^−^^1^
*c* = 11.938 (3) Å	*T* = 93 K
β = 90.265 (3)°	Block, colorless
*V* = 1117.9 (4) Å^3^	0.40 × 0.40 × 0.35 mm
*Z* = 4	

### Data collection

**Table:**

Rigaku SPIDER diffractometer	2532 independent reflections
Radiation source: Rotating Anode	2203 reflections with *I* > 2σ(*I*)
graphite	*R*_int_ = 0.031
ω scans	θ_max_ = 27.5°, θ_min_ = 3.0°
Absorption correction: multi-scan (*RAPID-AUTO*; Rigaku, 2004)	*h* = −13→14
*T*_min_ = 0.880, *T*_max_ = 0.894	*k* = −10→10
8869 measured reflections	*l* = −15→14

### Refinement

**Table:**

Refinement on *F*^2^	Primary atom site location: structure-invariant direct methods
Least-squares matrix: full	Secondary atom site location: difference Fourier map
*R*[*F*^2^ > 2σ(*F*^2^)] = 0.036	Hydrogen site location: inferred from neighbouring sites
*wR*(*F*^2^) = 0.099	H atoms treated by a mixture of independent and constrained refinement
*S* = 1.00	*w* = 1/[σ^2^(*F*_o_^2^) + (0.0582*P*)^2^ + 0.06*P*] where *P* = (*F*_o_^2^ + 2*F*_c_^2^)/3
2532 reflections	(Δ/σ)_max_ < 0.001
145 parameters	Δρ_max_ = 0.29 e Å^−^^3^
1 restraint	Δρ_min_ = −0.20 e Å^−^^3^

### Special details

**Table:**

Geometry. All e.s.d.'s (except the e.s.d. in the dihedral angle between two l.s. planes) are estimated using the full covariance matrix. The cell e.s.d.'s are taken into account individually in the estimation of e.s.d.'s in distances, angles and torsion angles; correlations between e.s.d.'s in cell parameters are only used when they are defined by crystal symmetry. An approximate (isotropic) treatment of cell e.s.d.'s is used for estimating e.s.d.'s involving l.s. planes.
Refinement. Refinement of *F*^2^ against ALL reflections. The weighted *R*-factor *wR* and goodness of fit *S* are based on *F*^2^, conventional *R*-factors *R* are based on *F*, with *F* set to zero for negative *F*^2^. The threshold expression of *F*^2^ > σ(*F*^2^) is used only for calculating *R*-factors(gt) *etc*. and is not relevant to the choice of reflections for refinement. *R*-factors based on *F*^2^ are statistically about twice as large as those based on *F*, and *R*- factors based on ALL data will be even larger.

### Fractional atomic coordinates and isotropic or equivalent isotropic displacement parameters (Å^2^)

**Table:**

	*x*	*y*	*z*	*U*_iso_*/*U*_eq_	
Cl1	0.19761 (3)	0.57092 (4)	0.31835 (3)	0.02509 (14)	
O1	0.88525 (10)	0.95264 (13)	0.14174 (10)	0.0295 (3)	
O2	0.79630 (9)	0.72040 (13)	0.17923 (9)	0.0273 (3)	
O3	0.40590 (10)	0.32662 (16)	0.33959 (12)	0.0382 (3)	
N1	0.42391 (11)	0.70595 (14)	0.18900 (10)	0.0184 (3)	
N2	0.57147 (10)	0.84373 (14)	0.12622 (10)	0.0184 (3)	
C1	0.42085 (12)	0.68984 (18)	0.07348 (12)	0.0210 (3)	
C2	0.51409 (12)	0.77535 (17)	0.03442 (12)	0.0211 (3)	
H2	0.5366	0.7869	−0.0418	0.025*	
C3	0.51465 (12)	0.79974 (16)	0.21927 (12)	0.0178 (3)	
C4	0.32597 (14)	0.5958 (2)	0.01537 (14)	0.0292 (4)	
H4A	0.3426	0.5934	−0.0652	0.035*	
H4B	0.2469	0.6445	0.0281	0.035*	
H4C	0.3258	0.4877	0.0449	0.035*	
C5	0.54916 (13)	0.84647 (19)	0.33464 (12)	0.0234 (3)	
H5A	0.5677	0.9610	0.3355	0.028*	
H5B	0.6236	0.7891	0.3562	0.028*	
C6	0.45261 (15)	0.8131 (2)	0.42068 (13)	0.0304 (4)	
H6A	0.3789	0.8710	0.4008	0.037*	
H6B	0.4809	0.8473	0.4947	0.037*	
H6C	0.4355	0.6994	0.4222	0.037*	
C7	0.67760 (12)	0.94450 (17)	0.12097 (12)	0.0202 (3)	
H7A	0.6672	1.0345	0.1731	0.024*	
H7B	0.6849	0.9879	0.0443	0.024*	
C8	0.79233 (12)	0.85727 (18)	0.15091 (12)	0.0205 (3)	
H1N	0.3693 (15)	0.667 (2)	0.2348 (14)	0.027 (4)*	
H1O	0.961 (2)	0.900 (3)	0.1558 (18)	0.060 (7)*	
H3A	0.344 (2)	0.397 (3)	0.336 (2)	0.077 (8)*	
H3B	0.385 (2)	0.243 (3)	0.289 (2)	0.067 (7)*	

### Atomic displacement parameters (Å^2^)

**Table:**

	*U*^11^	*U*^22^	*U*^33^	*U*^12^	*U*^13^	*U*^23^
Cl1	0.0190 (2)	0.0276 (2)	0.0287 (2)	−0.00309 (14)	0.00545 (15)	−0.00028 (14)
O1	0.0168 (5)	0.0245 (6)	0.0472 (7)	−0.0020 (5)	0.0009 (5)	0.0008 (5)
O2	0.0199 (5)	0.0236 (6)	0.0384 (6)	0.0035 (4)	0.0019 (5)	0.0087 (5)
O3	0.0206 (6)	0.0333 (7)	0.0605 (9)	0.0012 (5)	−0.0053 (6)	−0.0059 (6)
N1	0.0141 (6)	0.0215 (6)	0.0196 (6)	−0.0007 (5)	0.0018 (5)	−0.0005 (5)
N2	0.0139 (6)	0.0212 (6)	0.0202 (6)	0.0015 (5)	0.0002 (5)	0.0007 (5)
C1	0.0162 (7)	0.0250 (8)	0.0220 (7)	0.0033 (6)	−0.0002 (6)	−0.0028 (6)
C2	0.0178 (7)	0.0276 (8)	0.0178 (7)	0.0040 (6)	0.0004 (5)	−0.0008 (6)
C3	0.0139 (6)	0.0179 (7)	0.0217 (7)	0.0029 (5)	0.0018 (5)	−0.0005 (5)
C4	0.0202 (8)	0.0392 (10)	0.0281 (8)	−0.0016 (7)	−0.0013 (6)	−0.0088 (7)
C5	0.0236 (8)	0.0269 (8)	0.0197 (7)	−0.0040 (6)	−0.0004 (6)	−0.0027 (6)
C6	0.0303 (9)	0.0381 (10)	0.0229 (8)	−0.0054 (7)	0.0031 (7)	−0.0040 (7)
C7	0.0165 (7)	0.0195 (7)	0.0247 (7)	−0.0007 (6)	0.0021 (6)	0.0018 (5)
C8	0.0173 (7)	0.0232 (8)	0.0211 (7)	−0.0001 (6)	0.0021 (6)	−0.0003 (6)

### Geometric parameters (Å, °)

**Table:**

O1—C8	1.3125 (18)	C3—C5	1.481 (2)
O1—H1O	0.96 (2)	C4—H4A	0.9800
O2—C8	1.2063 (18)	C4—H4B	0.9800
O3—H3A	0.91 (2)	C4—H4C	0.9800
O3—H3B	0.96 (2)	C5—C6	1.513 (2)
N1—C3	1.3290 (18)	C5—H5A	0.9900
N1—C1	1.3860 (18)	C5—H5B	0.9900
N1—H1N	0.881 (17)	C6—H6A	0.9800
N2—C3	1.3322 (18)	C6—H6B	0.9800
N2—C2	1.3903 (18)	C6—H6C	0.9800
N2—C7	1.4534 (18)	C7—C8	1.5109 (19)
C1—C2	1.346 (2)	C7—H7A	0.9900
C1—C4	1.487 (2)	C7—H7B	0.9900
C2—H2	0.9500		
			
C8—O1—H1O	112.3 (13)	H4B—C4—H4C	109.5
H3A—O3—H3B	106.1 (16)	C3—C5—C6	113.66 (12)
C3—N1—C1	110.13 (12)	C3—C5—H5A	108.8
C3—N1—H1N	125.1 (11)	C6—C5—H5A	108.8
C1—N1—H1N	124.5 (11)	C3—C5—H5B	108.8
C3—N2—C2	108.97 (12)	C6—C5—H5B	108.8
C3—N2—C7	125.80 (12)	H5A—C5—H5B	107.7
C2—N2—C7	125.22 (12)	C5—C6—H6A	109.5
C2—C1—N1	106.06 (12)	C5—C6—H6B	109.5
C2—C1—C4	131.87 (14)	H6A—C6—H6B	109.5
N1—C1—C4	122.06 (13)	C5—C6—H6C	109.5
C1—C2—N2	107.40 (13)	H6A—C6—H6C	109.5
C1—C2—H2	126.3	H6B—C6—H6C	109.5
N2—C2—H2	126.3	N2—C7—C8	112.54 (12)
N1—C3—N2	107.43 (12)	N2—C7—H7A	109.1
N1—C3—C5	127.13 (13)	C8—C7—H7A	109.1
N2—C3—C5	125.44 (13)	N2—C7—H7B	109.1
C1—C4—H4A	109.5	C8—C7—H7B	109.1
C1—C4—H4B	109.5	H7A—C7—H7B	107.8
H4A—C4—H4B	109.5	O2—C8—O1	125.81 (14)
C1—C4—H4C	109.5	O2—C8—C7	124.34 (13)
H4A—C4—H4C	109.5	O1—C8—C7	109.85 (13)
			
C3—N1—C1—C2	0.88 (16)	C7—N2—C3—N1	−178.83 (12)
C3—N1—C1—C4	−177.84 (13)	C2—N2—C3—C5	179.31 (13)
N1—C1—C2—N2	−0.76 (16)	C7—N2—C3—C5	0.3 (2)
C4—C1—C2—N2	177.78 (15)	N1—C3—C5—C6	−15.1 (2)
C3—N2—C2—C1	0.40 (16)	N2—C3—C5—C6	165.88 (14)
C7—N2—C2—C1	179.39 (13)	C3—N2—C7—C8	77.86 (17)
C1—N1—C3—N2	−0.63 (16)	C2—N2—C7—C8	−100.96 (15)
C1—N1—C3—C5	−179.78 (14)	N2—C7—C8—O2	−2.0 (2)
C2—N2—C3—N1	0.14 (15)	N2—C7—C8—O1	178.25 (12)

### Hydrogen-bond geometry (Å, °)

**Table:**

*D*—H···*A*	*D*—H	H···*A*	*D*···*A*	*D*—H···*A*
N1—H1N···Cl1	0.881 (17)	2.300 (18)	3.1635 (14)	166.6 (15)
O3—H3A···Cl1	0.91 (2)	2.20 (2)	3.1062 (14)	177 (2)
O1—H1O···O3^i^	0.96 (2)	1.60 (2)	2.5557 (16)	170 (2)
O3—H3B···Cl1^ii^	0.96 (2)	2.14 (2)	3.0860 (14)	168 (2)

Symmetry codes: (i) −*x*+3/2, *y*+1/2, −*z*+1/2; (ii) −*x*+1/2, *y*−1/2, −*z*+1/2.
